# UBE2N as a novel prognostic and therapeutic biomarker of lung adenocarcinoma

**DOI:** 10.3389/fimmu.2025.1636503

**Published:** 2025-08-11

**Authors:** Haofeng Yin, Yibo Xue, Chen Wang, Yanqin Wu, Yuchen Guo, Chunzhen Li, Yunyan Zhang, Shulei Yin, Tiejun Zhao

**Affiliations:** ^1^ Institute of Immunology, School of Basic Medicine, Naval Medical University, Shanghai, China; ^2^ Department of Thoracic Surgery, Changhai Hospital, Naval Medical University, Shanghai, China; ^3^ Department of Respiratory and Critical Care Medicine, Changzheng Hospital, Naval Medical University, Shanghai, China

**Keywords:** lung adenocarcinoma, UBE2N, prognosis, biomarker, immunotherapy

## Abstract

**Background:**

Lung adenocarcinoma (LUAD) represents a significant global health burden. The absence of reliable biomarkers and the heterogeneity in treatment responses continue to hinder improvements in patient prognosis. This study aimed to identify novel biomarkers capable of predicting patient outcomes and therapeutic responsiveness, while also assessing their potential as intervention targets for LUAD.

**Methods:**

Multiple cohorts from public databases were employed to screen key prognostic genes, followed by external validations. Clinicopathological indicators were integrated to analyze the independent prognostic role of the key gene UBE2N and its association with LUAD progression. Functional enrichment analysis elucidated the biological mechanisms regulated by UBE2N. Differences in immune microenvironment components, immunoregulatory gene expression, and immune functional activities between subgroups stratified by UBE2N expression levels. The role of UBE2N in predicting tumor therapeutic susceptibility was characterized using bioinformatics algorithms combined with publicly available CRISPR screening datasets and immunotherapy cohorts. Immunohistochemistry, cell viability, and apoptosis experiments were conducted to verify the oncogenic effects of UBE2N.

**Results:**

UBE2N was identified as an independent prognostic biomarker for LUAD. Elevated UBE2N expression correlated with poorer patient survival rates and advanced disease stages. Genes associated with UBE2N were significantly enriched in critical cellular processes, including DNA replication, nucleosome assembly, and neutrophil extracellular trap formation. High-UBE2N tumors exhibited enhanced cell cycle, DNA replication, and oxidative phosphorylation activities. Low-UBE2N tumors exhibit elevated proportions of intratumoral NK cells, dendritic cells, effector T cells, and enhanced antigen processing and presentation. UBE2N was a potential promoter of immune evasion and drug resistance, with its high expression suggestive of low responsiveness to cancer immunotherapy and targeted therapies. Three potential UBE2N-inhibiting compounds were identified. Tissue microarrays confirmed UBE2N overexpression in LUAD, correlating with tumor size, while UBE2N knockdown suppressed tumor cell viability and induced apoptosis.

**Conclusions:**

UBE2N may serve as a promising prognostic biomarker and therapeutic target for LUAD. Inhibition of UBE2N is expected to suppress LUAD progression and enhance therapeutic efficacy.

## 1 Introduction

The 2024 global cancer statistics revealed lung cancer as the most commonly diagnosed malignancy, with close to 2.5 million new cases reported, representing roughly 12.5% of all cancer diagnoses worldwide (1, 2). Among its subtypes, lung adenocarcinoma (LUAD) is the most prevalent subtype of non-small cell lung cancer (NSCLC) (3, 4). Due to the absence of distinct early symptoms, a large number of patients cannot be diagnosed at an early stage, posing significant challenges for treatment and prognosis (5–7). Despite recent advancements in detection methods and targeted therapies, the therapeutic outlook for LUAD remains poor, with five-year survival rates remaining at approximately 15% (8). Recently, immune checkpoint blockade (ICB) therapy has revolutionized NSCLC management (9). These therapies work by inhibiting key immunosuppressive signals, such as PD-1/PD-L1, to boost the anti-tumor immunity and achieve long-term remission in some patients (10). Nevertheless, their widespread application is hindered by limited response rates and a scarcity of predictive biomarkers (9). Consequently, there is a critical need to uncover novel and reliable biomarkers capable of accurately predicting patient prognosis and immunotherapy response.

Viral infections, such as those caused by HBV, HPV, and EBV, can lead to human cancer by manipulating the biological processes of host cells (11). These include integrating viral genes, regulating tumor suppressor and oncogenes, inducing genomic instability, disrupting cell cycles and apoptosis, evading immune responses, promoting chronic inflammation and oxidative stress, remodeling the extracellular matrix, facilitating angiogenesis, and reprogramming metabolism (12–19). Therefore, based on the molecular mechanisms of antiviral immunity, the identification of key molecules with the potential to predict tumor occurrence, treatment, and prognosis represents a highly promising research direction. This endeavor will provide novel insights into deciphering the interplay between tumor immunity and innate immunity.

Ubiquitination, a key post-translational modification (PTM), fundamentally regulates multiple cellular processes. This enzymatic cascade requires three primary components: E1 ubiquitin-activating enzymes, E2 ubiquitin-conjugating enzymes, and E3 ubiquitin ligases (20). Recent studies highlight the critical role of E2 enzymes in tumor progression and immune regulation, making them potential biomarkers for prognosis and targets for immunotherapy (21–23). In NSCLC, elevated UBE2S levels enhance cellular proliferation, migration, and stem-like properties, indicating its association with unfavorable clinical outcomes (24). Similarly, UBE2C expression has been linked to immune cell infiltration, potentially influencing immunotherapy efficacy (25, 26). And UBE2N (also known as UBC13), another member of the E2 enzyme family, was characterized as a potential cancer target in the present study. Multiple studies have demonstrated that UBE2N contributes to DNA repair mechanisms through forming complexes with UEV proteins (such as Mms2) to assemble K63-linked polyubiquitin chains, which can promote cell proliferation (27, 28). Additionally, researchers have confirmed UBE2N as a potential prognostic indicator, with its expression profile correlating significantly with clinical outcomes and therapeutic responses in melanoma, breast carcinoma, and neuroblastoma (29–31). Nevertheless, its specific contributions to lung adenocarcinoma (LUAD) pathogenesis, tumor immunity, and therapeutic response remain poorly characterized.

This study aims to identify key molecules critically involved in the prognosis and immunotherapy response of LUAD. Through comprehensive multi-cohort screening, we discovered that UBE2N acts as an oncogenic driver, accelerating disease progression, remodeling the tumor immune microenvironment, and conferring resistance to both immunotherapy and chemotherapy in LUAD. Our findings demonstrate that UBE2N represents a robust biomarker with significant associations with patient prognosis, disease progression, tumor microenvironment characteristics, and treatment sensitivity in LUAD. Collectively, these findings implicate UBE2N as a potential therapeutic target for the development of treatment modalities and the facilitation of precision medicine in LUAD.

## 2 Materials and methods

### 2.1 Data sources and processing

We retrieved transcriptomic and clinicopathologic phenotype data of LUAD cohorts for gene screening from two publicly available repositories: The Cancer Genome Atlas (TCGA, https://portal.gdc.cancer.gov/) and Gene Expression Omnibus (GEO, https://www.ncbi.nlm.nih.gov/geo). The transcriptomic data in transcripts per million (TPM) format and corresponding clinicopathologic phenotypes of the TCGA-LUAD cohort were retrieved from UCSC Xena. Expression matrices obtained from the GEO database had been normalized by the original researchers. Genes with extremely low expression levels (average expression value < 1) and those with missing values were further filtered out. Additionally, samples lacking complete clinical information were excluded from the analysis. No dataset merging was performed in the present study, so batch correction was not involved. **Supplementary Table S1** lists the basic information and analytical usage of the datasets used in this study. The antiviral-related gene set was obtained from the MsigDB database (32).

### 2.2 Screening of key prognostic genes and survival analysis

By integrating gene expression and survival data from multiple datasets, we conducted univariable Cox regression analysis and retained overlapping genes with significant prognostic relevance in LUAD (P < 0.05). Given the close association between viral infections, host antiviral immune responses, and lung cancer progression, we further extracted key prognostic genes related to antiviral immunity (33, 34). Subsequently, patients were stratified into high- and low-expression subgroups based on median gene expression values, followed by survival analyses across distinct cohorts.

### 2.3 Clinicopathological relevance analysis

The independent prognostic power of UBE2N was tested via univariate and multivariate Cox proportional hazards models. The clinical relevance of UBE2N was further explored by examining its relationships with pathological variables and comparing the expression of UBE2N in different clinicopathologic subgroups. Finally, we developed a prognostic nomogram to facilitate precise survival prediction in patients. To facilitate precise survival prediction, a nomogram integrating pathological variables and UBE2N expression was constructed.

### 2.4 Co-expression network construction

Pearson’s correlation coefficients were calculated to detect genes exhibiting co-expression patterns with UBE2N, followed by a heatmap visualization of these associations. Subsequently, the 50 most strongly correlated (both positively and negatively) genes were chosen to establish a protein-protein interaction (PPI) network with the use of the STRING platform.

### 2.5 Functional enrichment analysis

TCGA-LUAD samples were classified into high-UBE2N and low-UBE2N subgroups. The DESeq2 package was implemented to identify differentially expressed genes (DEGs), with protein-coding genes meeting |log2FC| > 1 and p-value < 0.05 thresholds defined as statistically significant. Subsequent functional annotation was performed using the clusterProfiler package, covering Gene Ontology (GO) terms and KEGG pathways (35). Gene sets for Gene Set Enrichment Analysis (GSEA) were retrieved from the MSigDB (32). All genes were ranked by their fold changes, and GSEA was further performed using the “org.Hs.eg.db” and “clusterProfiler” packages with 10,000 permutations.

### 2.6 Characterization of the tumor immune landscape

To reveal the immunologic significance of UBE2N, we quantified tumor-infiltrating immune cell (TIICs) proportions across samples stratified by gene expression levels using single-sample gene set enrichment analysis (ssGSEA). Tumor microenvironment scores and purity estimates were concurrently calculated (36). Furthermore, we assessed correlations between UBE2N and immunomodulators, with boxplots visualizing expression differences across different subgroups. We also systematically examined associations between UBE2N and immune functional signatures using the GSVA and IOBR packages (37, 38). **Supplementary Table S2** lists the information and analytical usage of the gene sets used in this study.

### 2.7 Immunotherapy responsiveness analysis

We investigated the relationship between key genes and immunotherapy response through an integrated approach combining *in vitro* and *in vivo* CRISPR screening, real-world immunotherapy cohorts, and immunotherapeutic sensitivity algorithms. The role of UBE2N in immune evasion was analyzed using three CRISPR screen datasets, including an *in vitro* tumor-immune cell co-culture screening, an *in vitro* targeted screening of immunoregulatory factors (MHC-I/PD-L1), and an *in vivo* CRISPR screen in syngeneic mouse tumor models (39–41). Immunotherapy sensitivity was evaluated using Immunophenoscore (IPS) and multiple real-world immunotherapy cohorts (42–45). Cohort survival analysis and expression profile comparison were conducted to reveal the association between UBE2N level, survival event, and treatment responsiveness in patients undergoing immunotherapy (46, 47).

### 2.8 Chemosensitivity assessment

To evaluate potential associations between UBE2N expression and chemosensitivity of LUAD, we employed computational approaches utilizing both the oncopredict package and the CMAP platform (48, 49). Tumor transcriptomic profiles were processed using the oncopredict package, applying GDSC2-derived predictive models to estimate treatment response probabilities. UBE2N-associated differential gene expression profiles were uploaded to the CMAP platform, identifying compounds showing inverse connectivity with UBE2N. Structures of the compounds were derived from the PubChem database.

### 2.9 Pathologic and cellular experiments to validate the oncogenic function of UBE2N

The expression and functional characteristics of UBE2N in LUAD were validated using tissue microarrays (TMAs) and *in vitro* experiments. A tissue microarray comprising 35 paired human LUAD and adjacent non-tumor tissues was obtained from Servicebio Technology (Wuhan, China). Monoclonal antibodies against UBE2N (A9257) were purchased from Abclonal (Wuhan, China). Immunohistochemical (IHC) staining was performed following previously established protocols, and positive staining was quantified using Aipathwell software (50). Images of publicly available IHC sections of LUAD and normal lung tissue were obtained from the Human Protein Atlas (HPA) database (51). The A549 cell line was cultured in F-12K medium (Gibco, Cat. 21127022) supplemented with 10% fetal bovine serum (FBS) at 37°C under a 5% CO_2_ atmosphere. For gene knockdown, siRNA transfection was conducted using Lipofectamine™ RNAiMAX Reagent (Thermo Fisher Scientific). Effects of UBE2N on cellular proliferation and apoptosis were examined using the CCK-8 assay and an Annexin V-based apoptosis detection kit. The siRNA and qPCR primer sequences for human UBE2N are provided in **Supplementary Table S3**.

### 2.10 Statistical analysis

Statistical analyses were performed using R software (version 4.1.2), GraphPad Prism (version 8.0), and complementary online tools (Kaplan-Meier Plotter, STRING, and Cmap). Survival outcomes were assessed via Kaplan-Meier analysis. Group comparisons were conducted using the Wilcoxon test or Student’s t-test for two groups, and one-way ANOVA or Kruskal-Wallis test for multiple groups. Pearson correlation analysis evaluated associations between variables. A two-sided p-value < 0.05 defined statistical significance.

## 3 Results

### 3.1 Multi-cohort screen identified key prognostic genes in LUAD

**Figure 1:**
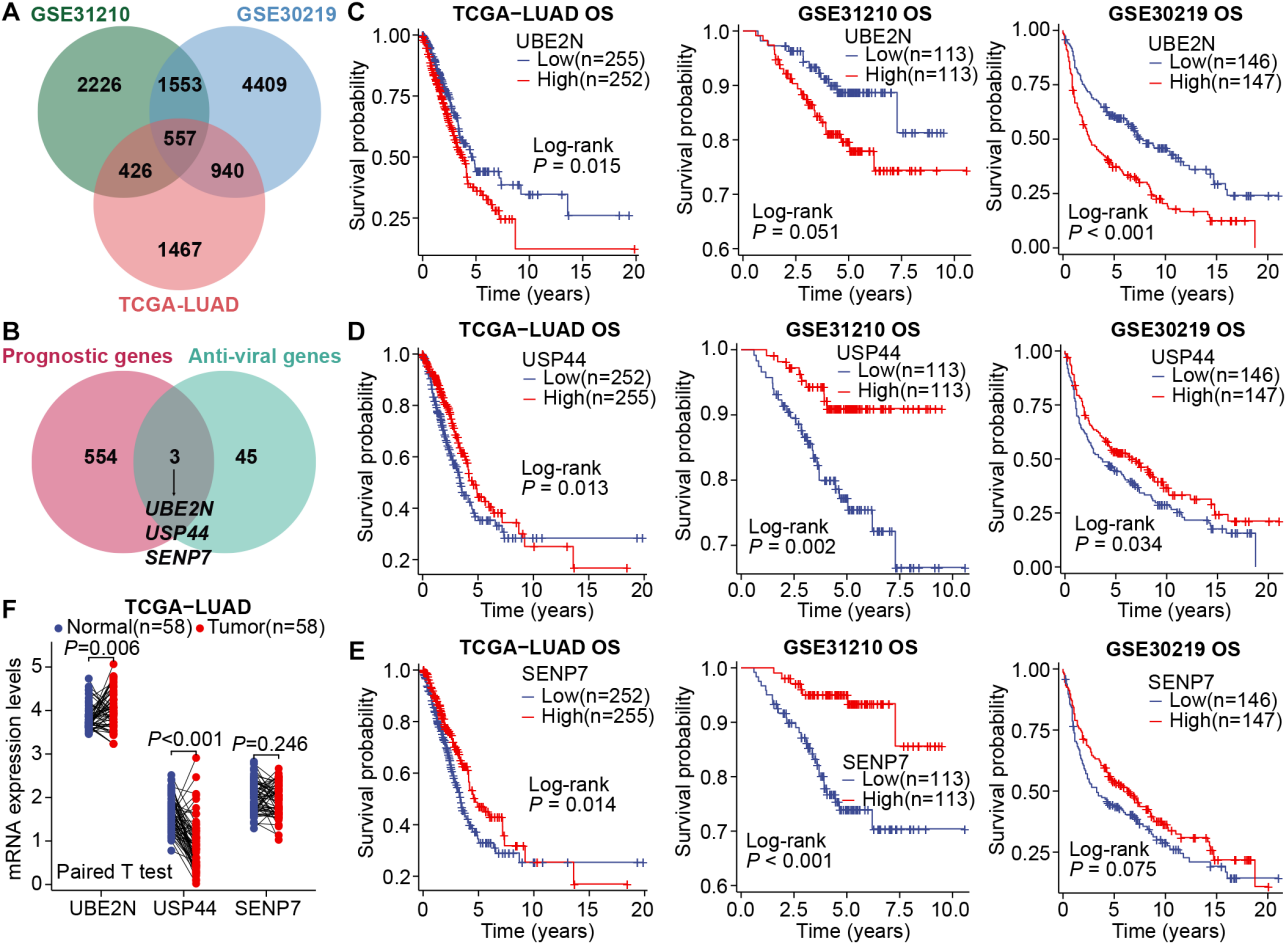
Screening of key prognostic genes in LUAD. **(A)** The Venn diagram for intersecting prognostic genes. **(B)** The Venn diagram for intersecting common prognostic genes and anti-viral genes. **(C–E)** Survival curves generated by stratifying patients based on the median expression levels of key genes, UBE2N **(C)**, USP44 **(D)**, and SENP7 **(E)**. **(F)** Differential expression of key genes in LUAD and paired paracancerous tissues.

To uncover key genes with robust prognostic effects in LUAD, we first utilized a multicohort-based univariate Cox regression screen. There were 2226, 4409, and 1467 candidate genes with prognostic effects in GSE31210, GSE30219, and TCGA-LUAD datasets, respectively. Taking the intersection of the above three gene groups, 557 key prognostic genes were initially identified (**Figure 1A**). Considering the complex roles of viral infection and antiviral immune mechanisms in the pathogenesis of various cancers, including NSCLC, we further extracted three key antiviral-related prognostic genes (UBE2N, USP44, and SENP7) from those 557 candidate genes (**Figure 1B**). Subsequently, the association of these three key genes with patient prognosis was assessed in multiple LUAD cohorts, including TCGA-LUAD, GSE31210, and GSE30219. Interestingly, high expression of UBE2N is associated with unfavorable prognosis, while high expression of the other two genes (USP44 and SENP7) is associated with favorable prognosis (**Figures 1C–E**). Moreover, expression profiling analysis showed that the expression of UBE2N in LUAD tissues was significantly up-regulated compared with paired paracancerous tissues, while USP44 was significantly down-regulated, and SENP7 showed no differential expression (**Figure 1F**). Pan-cancer expression analyses also showed that UBE2N expression was upregulated in most cancers (**Supplementary Figures S1A, B**). In summary, UBE2N and USP44 emerged as promising prognostic genes in LUAD.

### 3.2 Validating the prognostic roles of UBE2N and USP44 in multiple external cohorts

**Figure 2:**
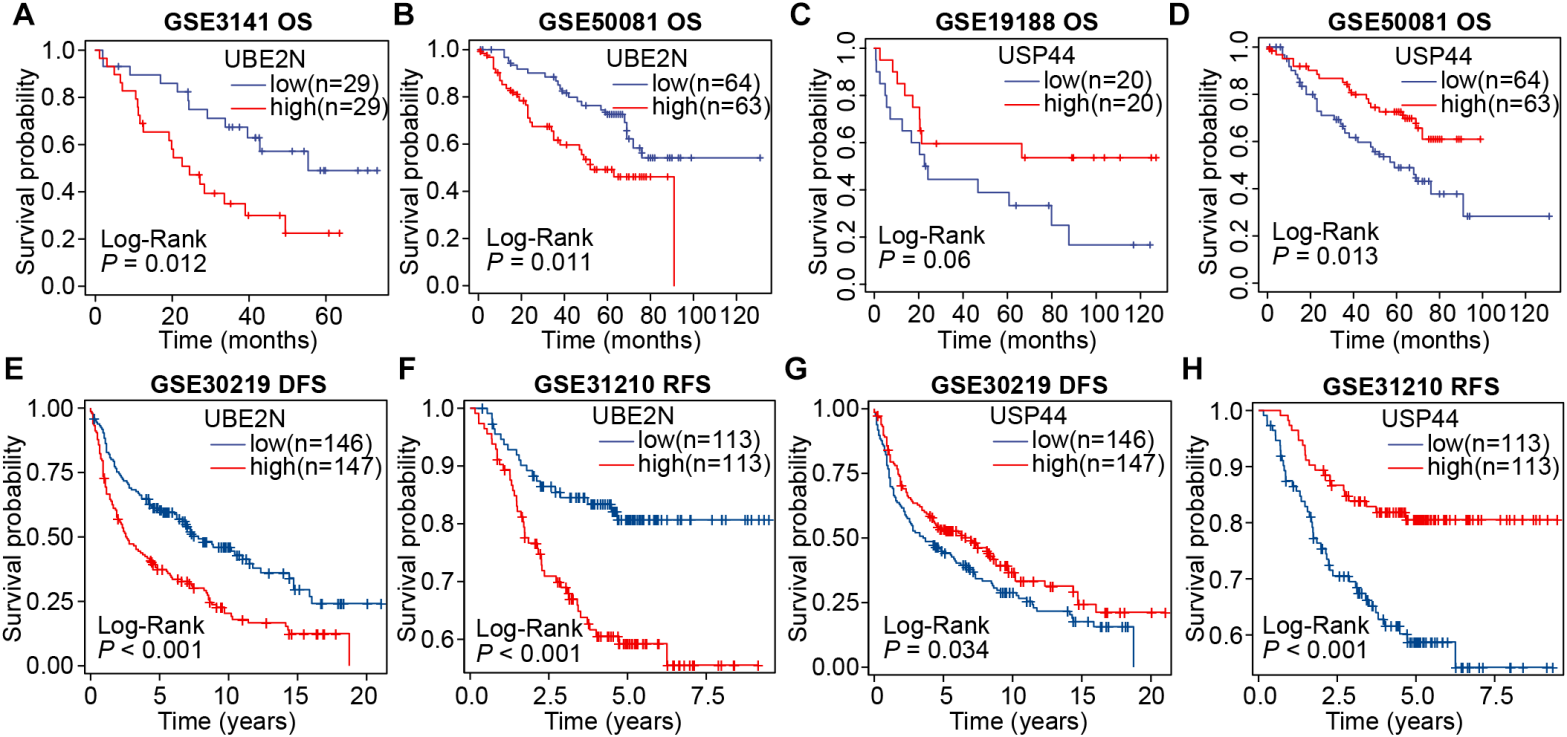
Validation of the prognostic roles of UBE2N and USP44 across multiple cohorts. **(A, B)** Survival curves were plotted by grouping patients according to UBE2N expression in the GSE3141 **(A)** and GSE50081 **(B)** cohorts. **(C, D)** Survival curves were plotted by grouping patients according to USP44 expression in the GSE19188 **(C)** and GSE50081 **(D)** cohorts. **(E, F)** DFS and RFS analyses based on UBE2N expression in the GSE30219 **(E)** and GSE31210 **(F)** cohorts. **(G, H)** DFS and RFS analyses based on USP44 expression in the GSE30219 **(G)** and GSE31210 **(H)** cohorts.

Subsequently, we validated the prognostic significance of UBE2N and USP44 using multiple external cohorts. Consistent with results from the aforementioned cohorts, elevated expression of UBE2N remained significantly associated with shorter overall survival in LUAD patients (*P* < 0.05; **Figures 2A, B**). Conversely, LUAD patients with high USP44 expression exhibited prolonged overall survival, although this association did not reach statistical significance in the GSE19188 dataset (*P* = 0.06 and *P* < 0.05; **Figures 2C, D**). Furthermore, we conducted additional analyses examining the relationship between gene expression and disease-free survival (DFS) as well as recurrence-free survival (RFS) in LUAD. The results demonstrated that high UBE2N expression was significantly correlated with both reduced DFS and RFS (**Figures 2E, F**). In contrast, USP44 showed an opposing prognostic pattern (**Figures 2G, H**). Collectively, through multi-cohort screening and validation, our study has identified UBE2N and USP44 as two crucial prognostic biomarkers in LUAD, with UBE2N emerging as a particularly robust predictor of unfavorable outcomes.

### 3.3 Identification of the independent prognostic role of UBE2N

**Figure 3:**
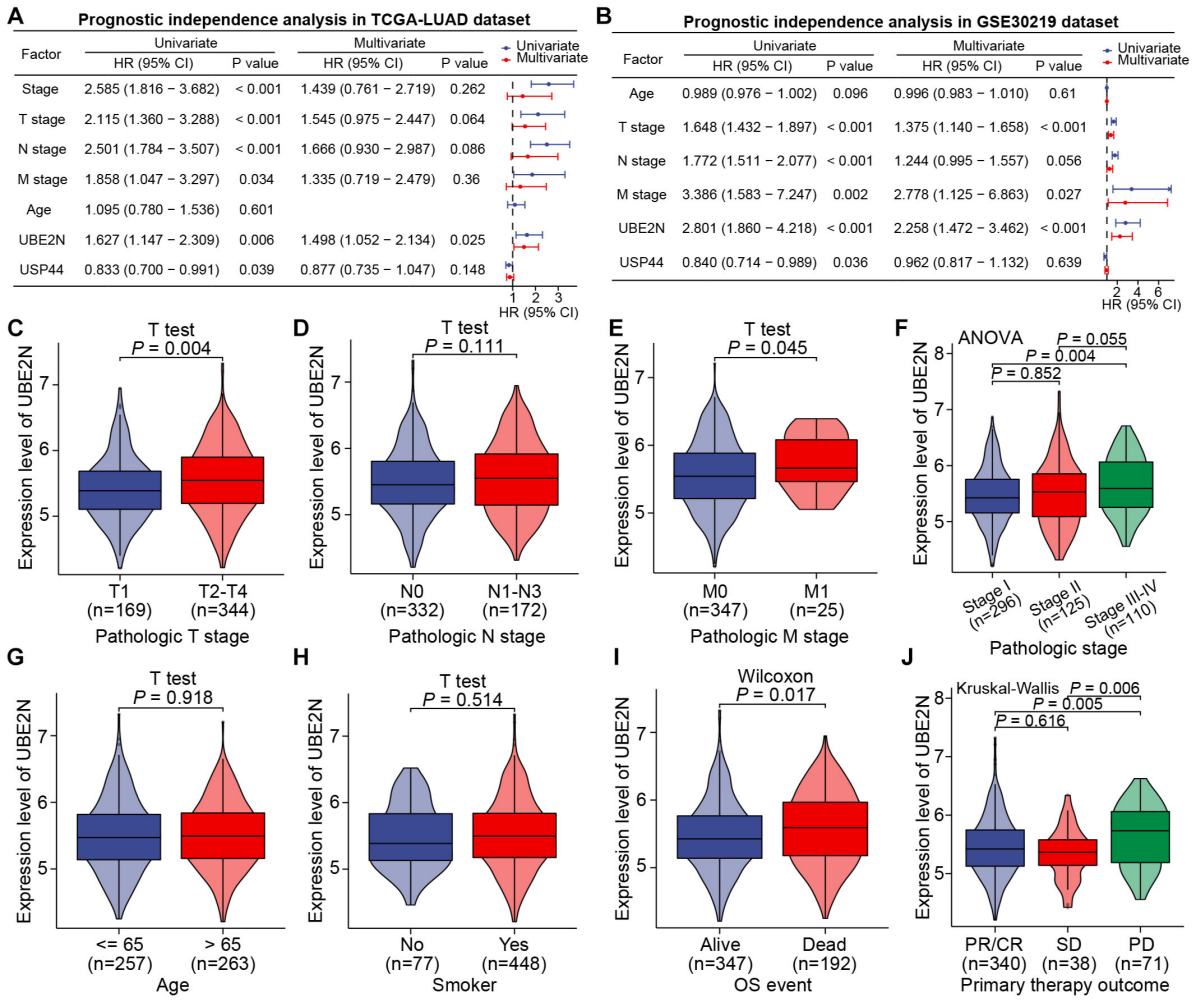
UBE2N was identified as an independent prognostic indicator of LUAD and correlates with clinicopathologic factors. **(A, B)** Prognostic independence analyses of key genes and clinicopathologic factors in TCGA-LUAD **(A)** and GSE30219 **(B)** cohorts. **(C–J)** Correlation between the expression of UBE2N and different clinicopathologic factors.

We then analyzed the independent prognostic utility of UBE2N and USP44, with UBE2N emerging as a robust, independent prognostic factor in the TCGA-LUAD cohort. And its association with disease progression remained statistically significant in the multivariate model, indicating minimal confounding by other clinical covariates (**Figure 3A**). The independent prognostic effect of UBE2N was then verified in the GSE30219 cohort (**Figure 3B**). However, while USP44 was identified as an independent indicator in univariate analysis, it failed to demonstrate independent prognostic significance in multivariate regression models in both cohorts (**Figures 3A, B**). Collectively, we focused our research on UBE2N, as analyses established UBE2N as a robust independent prognostic factor associated with adverse outcomes in LUAD.

### 3.4 UBE2N expression correlates with LUAD clinicopathological progression

The relationship between UBE2N and the clinicopathological progression of LUAD was further analyzed. Specifically, UBE2N expression was significantly elevated in tumors from patients with T2-T4 staging, stage II-IV disease, and M1 distant metastasis, but showed no significant correlation with nodal involvement based on the TCGA-LULAD cohort (**Figures 3C–F**). Furthermore, UBE2N expression patterns were independent of patient age or smoking status (**Figures 3G, H**). Notably, we observed marked upregulation of tumoral UBE2N in patients with adverse outcomes and progressive disease (PD) (**Figures 3J, K**).

**Figure 4:**
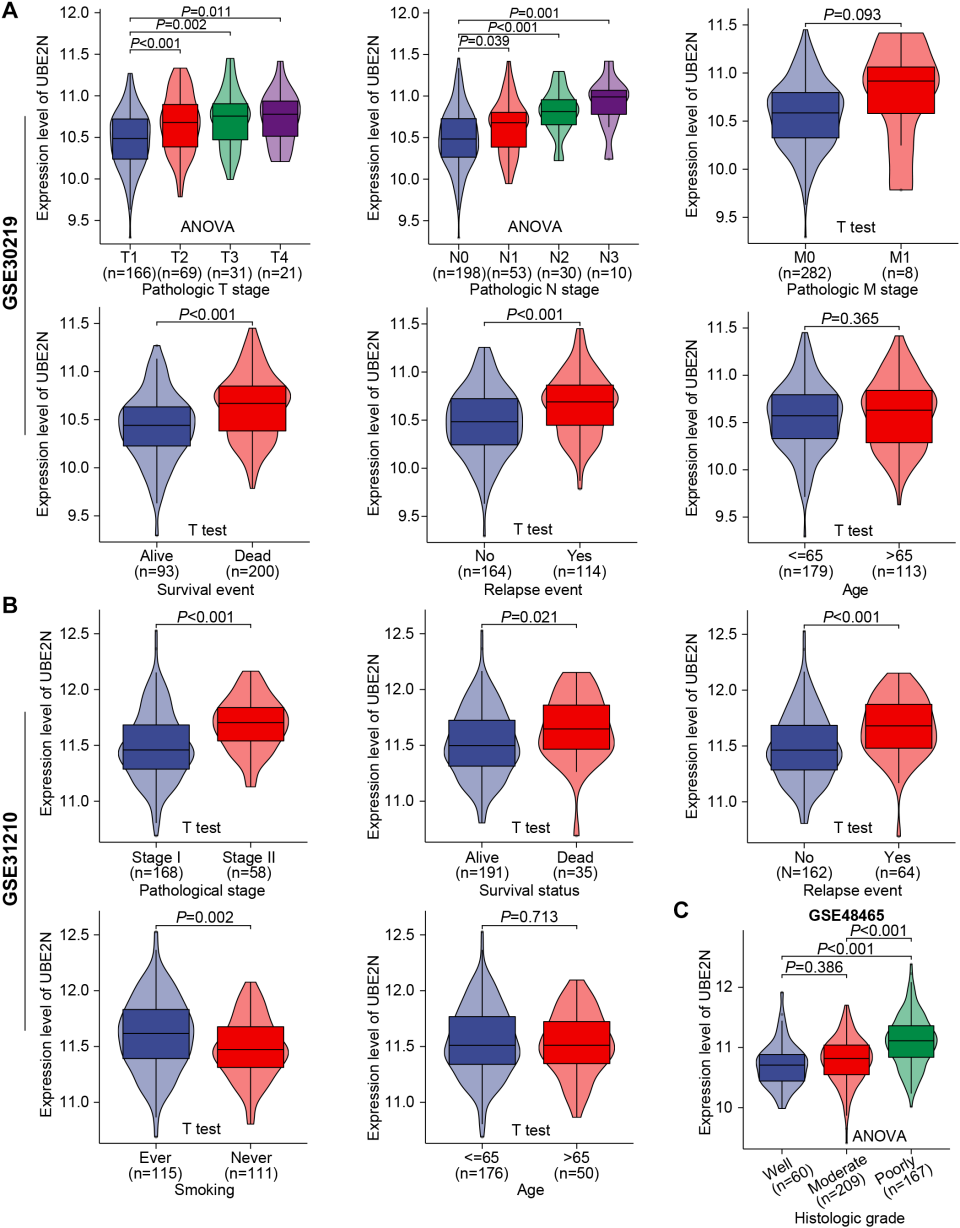
Validation of the association between UBE2N and the clinicopathological progression of LUAD in multiple cohorts. **(A, B)** Violin plots depict the correlation between UBE2N expression and clinical pathological parameters in cohorts GSE30219 **(A)** and GSE31210 **(B)**. **(C)** Expression levels of UBE2N in histologic grades with different degrees of differentiation in the GSE48465 dataset.

Further validating analyses were conducted in GSE30219, GSE31210, GSE48465, and GSE50081 cohorts. We confirmed the independence of UBE2N expression pattern from patient age. Moreover, a significant progressive increase in UBE2N expression was observed corresponding to advancing T-stage, N-stage, and overall stage (**Figures 4A, B**). In cohorts GSE48465 and GSE50081, UBE2N also exhibited a tendency toward upregulation in tumors with advanced T-stages (**Supplementary Figures S2A, B**). While UBE2N expression appeared elevated in tumors with distant metastasis, this difference did not reach statistical significance (*P* = 0.093, **Figure 4A**). Most importantly, UBE2N showed strong clinical relevance to LUAD progression and recurrence, demonstrating significant upregulation in tumors from patients who experienced mortality or disease recurrence compared to event-free survivors (**Figures 4A, B**). In addition, UBE2N expression levels progressively decreased with advancing histologic grade in the GSE48465 cohort, highlighting its potential inverse association with tumor differentiation status (**Figure 4C**). These results further verified the close correlation between UBE2N and the development, progression, and occurrence of LUAD. Therefore, we further integrated UBE2N and other clinicopathologic indicators collectively to develop a prognostic nomogram for accurate survival prediction (**Supplementary Figure S2C**).

### 3.5 UBE2N-related gene regulatory network and functional pathways

**Figure 5:**
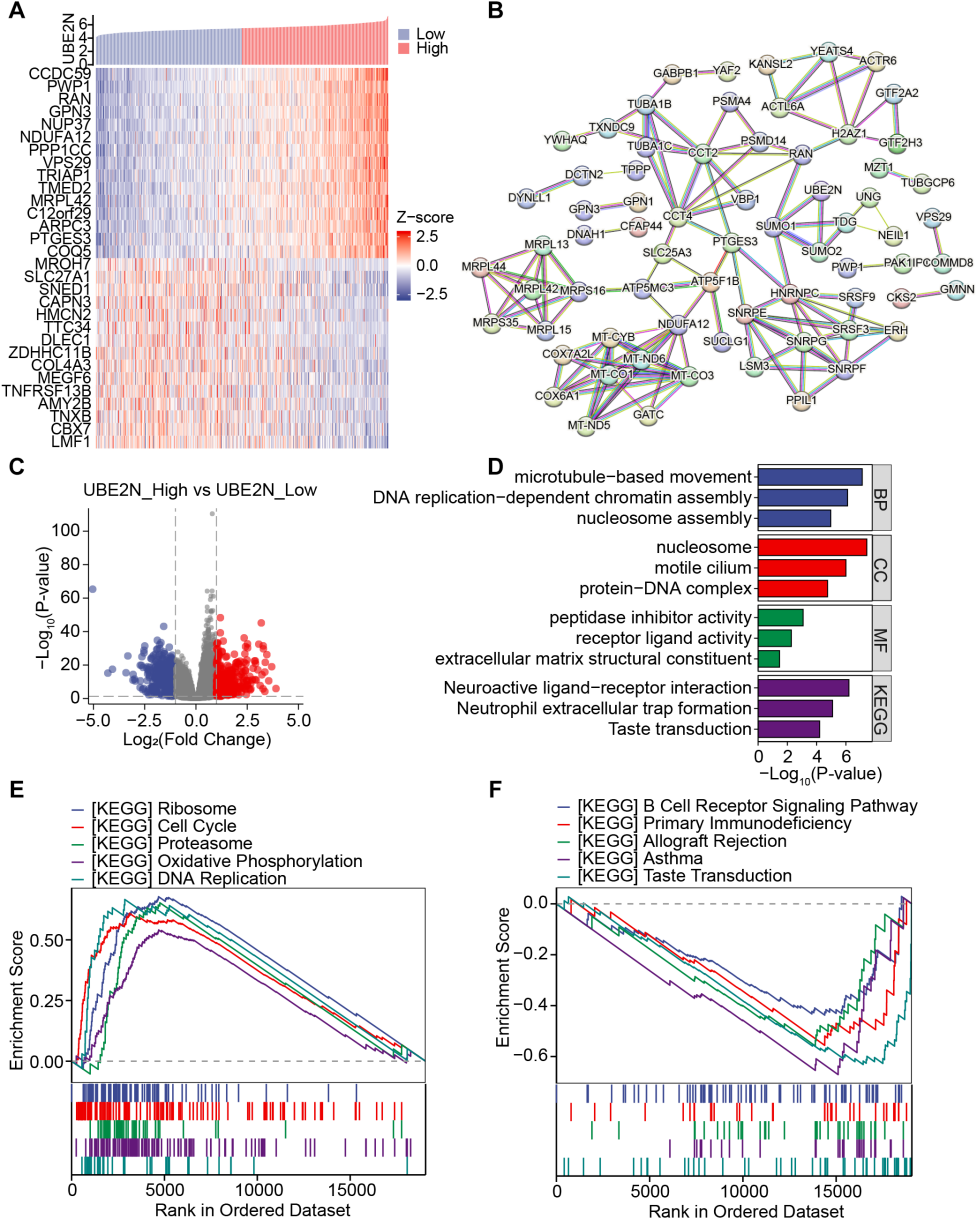
Investigation of the co-expressed gene network and associated functional pathways of UBE2N. **(A)** Heatmap showing the expression of co-expressed genes of UBE2N. **(B)** PPI network involving UBE2N and its co-expressed genes. **(C)** Volcano plot of UBE2N-related differential genes. **(D)** Functional enrichment analysis of UBE2N-related differential genes. **(E, F)** KEGG pathways enriched in high-UBE2N **(E)** and low-UBE2N groups **(F)**.

To analyze the molecular mechanism by which UBE2N promotes tumor progression, we screened its co-expressed genes and UBE2N-related DEGs. Firstly, the molecules showing significant positive and negative correlations with UBE2N were screened. The top 15 genes positively (including CCDC59) or negatively (including MROH7) correlated with UBE2N were presented in the heat map (**Figure 5A**). Potential UBE2N protein regulatory networks were predicted using the STRING database to further investigate possible functional relationships of UBE2N in LUAD (**Figure 5B**).

UBE2N-related DEGs were also screened, and the volcano plot demonstrated 507 down-regulated and 424 up-regulated genes associated with UBE2N (**Figure 5C**). The enrichment results demonstrated that UBE2N-associated DEGs were enriched in GO terms including microtubule movement, DNA replication, nucleosome assembly, extracellular matrix structure, and receptor ligand activity (**Figure 5D**). Moreover, neuroactive ligand-receptor interaction and neutrophil extracellular trap formation pathways were significantly enriched (**Figure 5D**). These results suggest that UBE2N expression may be involved in the regulation of biological processes related to cell motility, DNA replication, extracellular matrix remodeling, and neutrophil extracellular traps (NETs) formation.

In addition, GSEA results showed that high-UBE2N tumors were predominantly enriched with pathways associated with ribosome, cell cycle, proteasome, oxidative phosphorylation, and DNA replication (**Figure 5E**). GSVA results also demonstrated higher activities of cell cycle, DNA replication, and DNA damage repair in high-UBE2N tumors (**Supplementary Figure S3A**). However, low-UBE2N tumors were strongly linked with immunity-related pathways such as B cell receptor signaling pathway, primary immunodeficiency, allograft rejection, and asthma (**Figure 5F**). These results indicate that UBE2N-mediated cancer progression may be attributable to cell cycle and DNA replication, immune response, and immune cells. Elevated UBE2N levels in LUAD might promote tumor proliferation and suppress anti-tumor immune response.

### 3.6 Characterization of tumor immune landscape in LUAD

**Figure 6:**
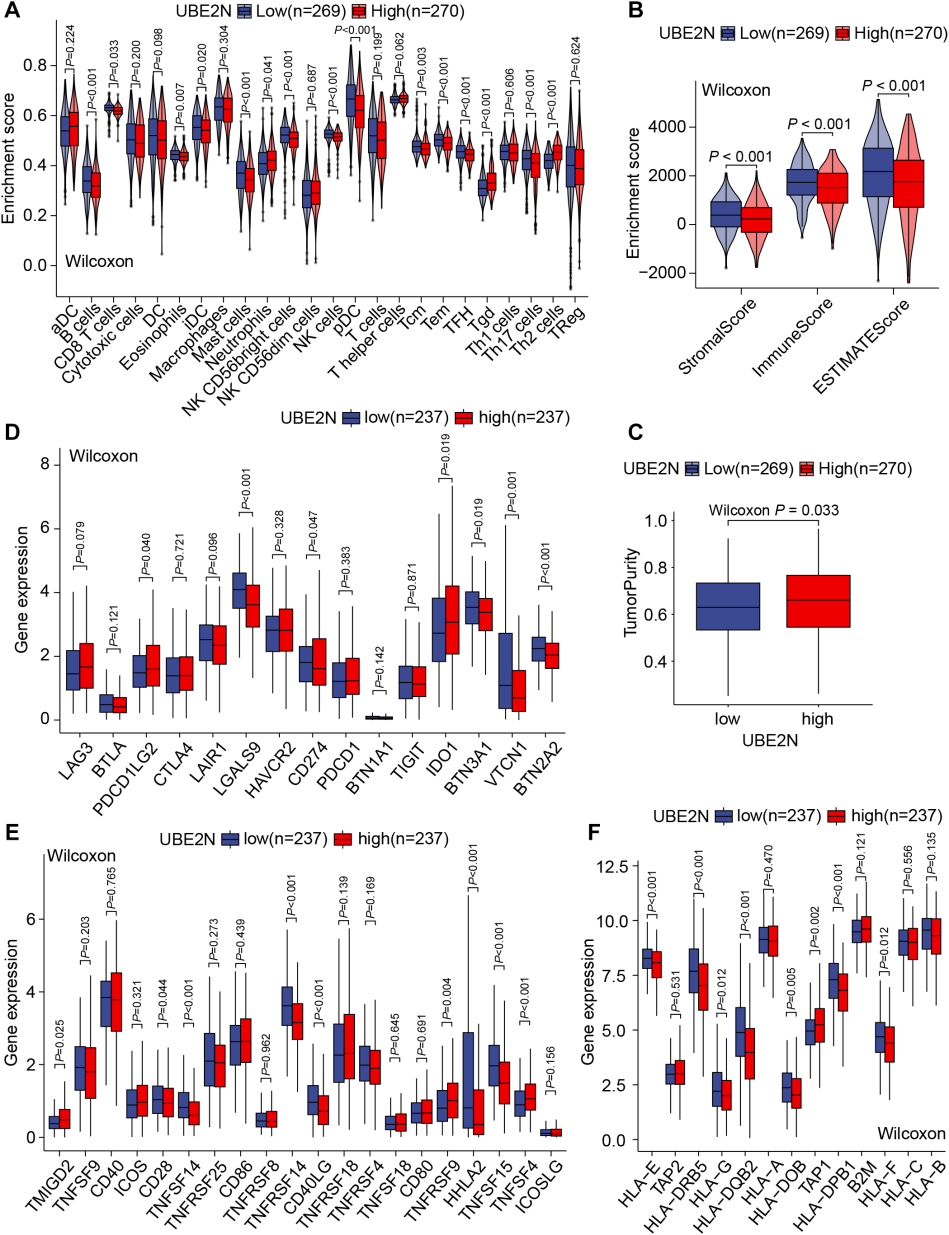
UBE2N-related TME and immune functional phenotypes. **(A)** The violin plot of tumor-infiltrating immune cell abundance. **(B)** The violin plot of TME scores. **(C)** The box plot of tumor purity. **(D–F)** Box plots of the expression of inhibitory immunomodulatory genes **(D)**, stimulatory immunomodulatory genes **(E)**, and genes related to antigen presentation and processing **(F)**.

The aforementioned results gave us a hint to explore the altered immune characteristics of tumors caused by UBE2N. Firstly, we analyzed the difference in TIIC abundance between low-UBE2N and high-UBE2N groups. Notably, the high-UBE2N tumors exhibited a higher level of neutrophils, γδT cells, and Th2 cells. While low-UBE2N tumors showed increased infiltrative B cells, CD8^+^ T cells, iDCs, mast cells, NK cells, pDCs, memory T cells, and T follicular helper (TFH) cells (**Figure 6A**). Similarly, the high-UBE2N group also exhibited elevated neutrophil and myeloid-derived suppressor cell (MDSC) signature scores but reduced T-cell signature scores (**Supplementary Figure S3B**). In addition, myeloid cells and chemokines associated with myeloid cell recruitment, especially CXCL8, showed a significant positive correlation with UBE2N. Thus, it is possible that UBE2N affects T cell infiltration and function via myeloid cells (**Supplementary Figures S3C–E**). Results based on the ESTIMATE algorithm showed the low-UBE2N group demonstrated significantly elevated stromal, immune, and ESTIMATE scores compared to the high-UBE2N group (**Figure 6B**). Consistently, the high-UBE2N group displayed markedly higher tumor purity (**Figure 6C**), further supporting the association between UBE2N expression, tumor progression, and immune suppression.

Apart from the association between UBE2N and TME (Tumor microenvironment) remodeling, we further hypothesized that UBE2N may regulate immune effector functions. Transcriptome analysis showed that the expression of immunomodulatory genes such as LAGLS9, CD274, VTCN1, BTN2A2, TNFSF14, TNFRSF14, CD40LG, and TNFSF15 was significantly up-regulated in low-UBE2N tumors as compared to high-UBE2N tumors, whereas the expression of PDCD1LG2, IDO1, TNFSF4, and TNFSF9 was significantly down-regulated (**Figures 6D, E**). In addition, multiple antigen-processing and presentation-related genes such as HLA-A, HLA-E, HLA-DRB5, and HLA-DQB2 were significantly upregulated in the low-UBE2N group (**Figure 6F**). Consistent results were also observed in GSVA analysis (**Supplementary Figure S3B**). These findings illustrated the involvement of UBE2N in the TME remodeling, mainly in its ability to limit intratumoral infiltration of effector immune cells and antigen processing and presentation.

### 3.7 UBE2N correlated with tumor immune evasion and immunotherapy resistance

**Figure 7:**
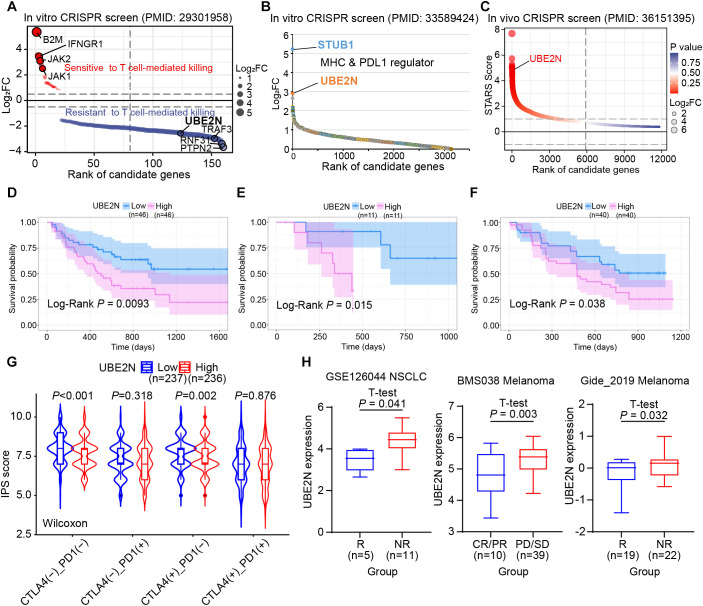
Role of UBE2N in tumor immune evasion and immunotherapy. **(A–C)** Publicly available CRISPR screen data reveal UBE2N as a promoter of tumor immune evasion. **(D–F)** Role of UBE2N expression in suggesting prognosis in cancer patients receiving immunotherapy. **(G)** IPS scores in UBE2N subgroups. **(H)** Role of UBE2N expression in predicting immunotherapy response in different cohorts.

Given the important role of UBE2N in mediating cancer progression and TME, we further integrated CRISPR screening, patient cohorts, and immune function algorithms to investigate the association between UBE2N and cancer immunotherapy responsiveness. The *in vitro* CRISPR screen showed that UBE2N knockdown promoted T cell-mediated tumor killing (**Figure 7A**). Another *in vitro* CRISPR screen also showed that UBE2N ranked highly among regulators of MHC-I and PD-L1 expression (**Figure 7B**). UBE2N was also identified as a potential tumor immune evasion promoter in an *in vivo* CRISPR screen (**Figure 7C**). Kaplan-Meier survival analysis across multiple cohorts demonstrated that cancer patients with low-UBE2N expression exhibited significantly prolonged survival compared to those with high-UBE2N expression when receiving immunotherapy. Low-UBE2N patients showed superior survival probability (**Figures 7D–F**). Consistent with our hypothesis, the low-UBE2N group demonstrated significantly higher IPS than the high-UBE2N group, independent of CTLA-4 expression patterns (**Figure 7G**). Analysis of UBE2N expression across immunotherapy cohorts revealed its significant role in predicting therapeutic outcomes. In the NSCLC and melanoma cohorts, patients in the immunotherapy-responsive group exhibited lower tumoral UBE2N expression (**Figure 7H**). Similarly, in another melanoma cohort, low UBE2N levels were associated with complete response (CR) or partial response (PR) of clinical treatment (**Figure 7H**). Moreover, ROC curves based on three cohorts showed that UBE2N expression performed well in assessing immunotherapy responsiveness (**Supplementary Figure S4A–C**). These findings suggest UBE2N as a promising biomarker for stratifying patients potentially benefiting from immunotherapy.

### 3.8 Value of UBE2N in predicting sensitivity to LUAD chemotherapy

**Figure 8:**
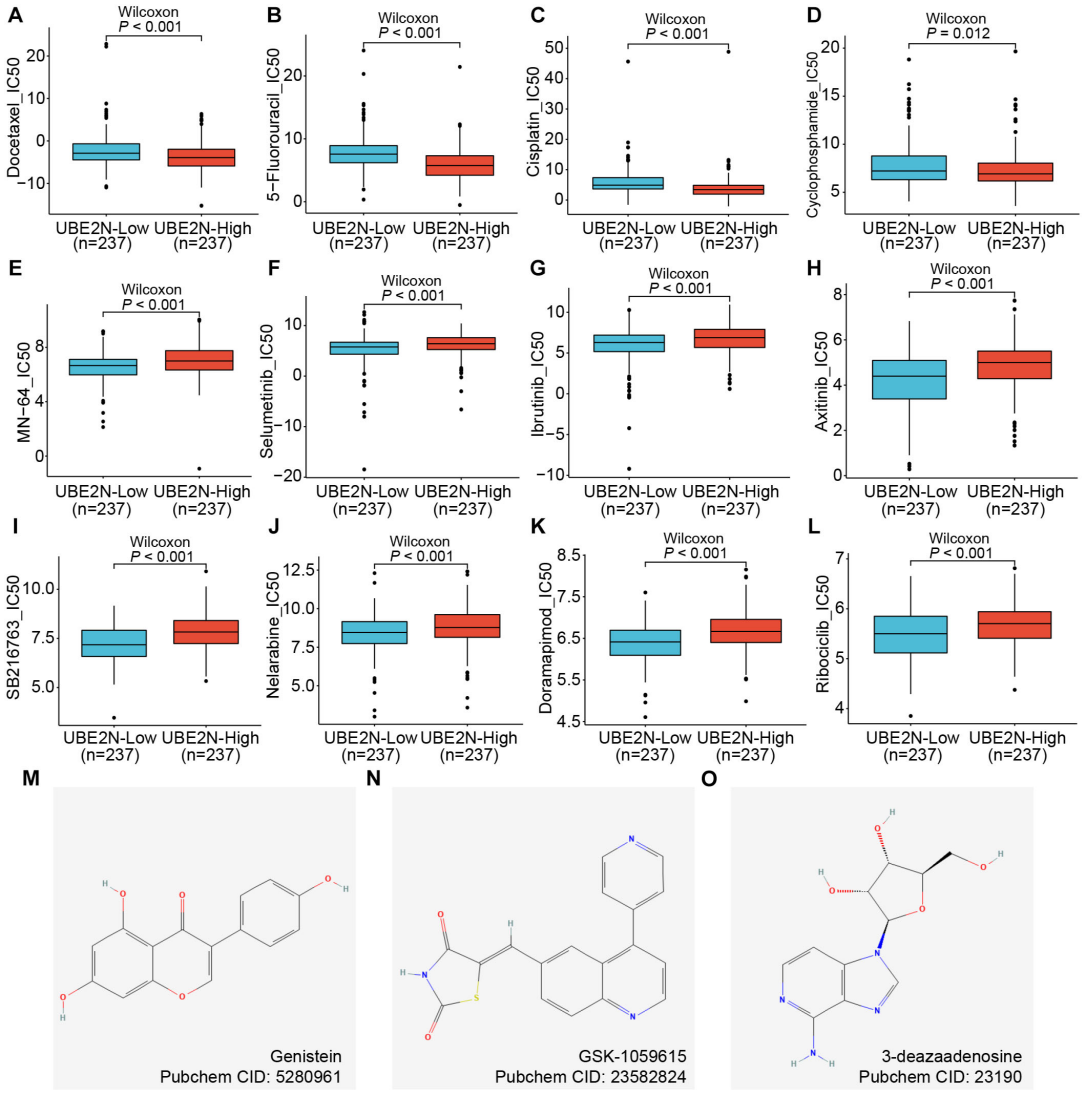
Role of UBE2N in cancer chemotherapy and targeted therapy. **(A–L)** Box plots of differences in drug sensitivity of patients in different UBE2N subgroups to chemotherapeutic and targeted agents including Docetaxel **(A)**, 5-Fluorouracil **(B)**, Cisplatin **(C)**, Cyclophosphamide **(D)**, MN-64 **(E)**, Selumetinib **(F)**, Ibrutinib **(G)**, Axitinib **(H)**, SB216763 **(I)**, Nelarabine **(J)**, Doramapimod **(K)**, and Ribociclib **(L)**. **(M–O)** Chemical structure diagrams of three potential compounds to inhibit UBE2N.

Chemotherapy remains a cornerstone of non-surgical management in LUAD. To evaluate UBE2N’s potential in stratifying chemotherapy response, we leveraged the oncoPredict algorithm to calculate drug sensitivity scores (IC50) across LUAD cohorts. Interestingly, higher IC50 values for common chemotherapeutic agents, including Docetaxel, 5-Fluorouracil, Cisplatin, and Cyclophosphamide, were observed in low-UBE2N tumors (**Figures 8A–D**). Notably, higher IC50 values directly correlate with increased drug resistance. This indicated that, although high expression of UBE2N is indeed associated with poor prognosis and immunosuppression, it also shows sensitivity to these four common chemotherapeutic drugs. However, the high-UBE2N tumors exhibited potential resistance to targeted agents such as MN-64, Ribociclib, Selumetinib, Ibrutinib, Axitinib, SB216763, and Doramapimod, as evidenced by the higher IC50 values in the high-UBE2N groups (**Figures 8E–L**). We further performed a connectivity mapping (CMap)-driven screening of potential drugs for targeting UBE2N. Three candidates (genistein, GSK-1059615, and 3-deazaadenosine) exhibited inverse transcriptomic alterations with UBE2N, and their chemical structures derived from the PubChem database were shown in **Figures 8M–Q**. We have listed the relevant information of the candidates in **Supplementary Table S4**. Notably, the inhibitory effects of genistein and 3-deazaadenosine on the growth of lung cancer have been reported, which corroborates our findings to some extent (52, 53).

### 3.9 Experimental validation of high UBE2N expression in LUAD and its oncogenic properties

**Figure 9:**
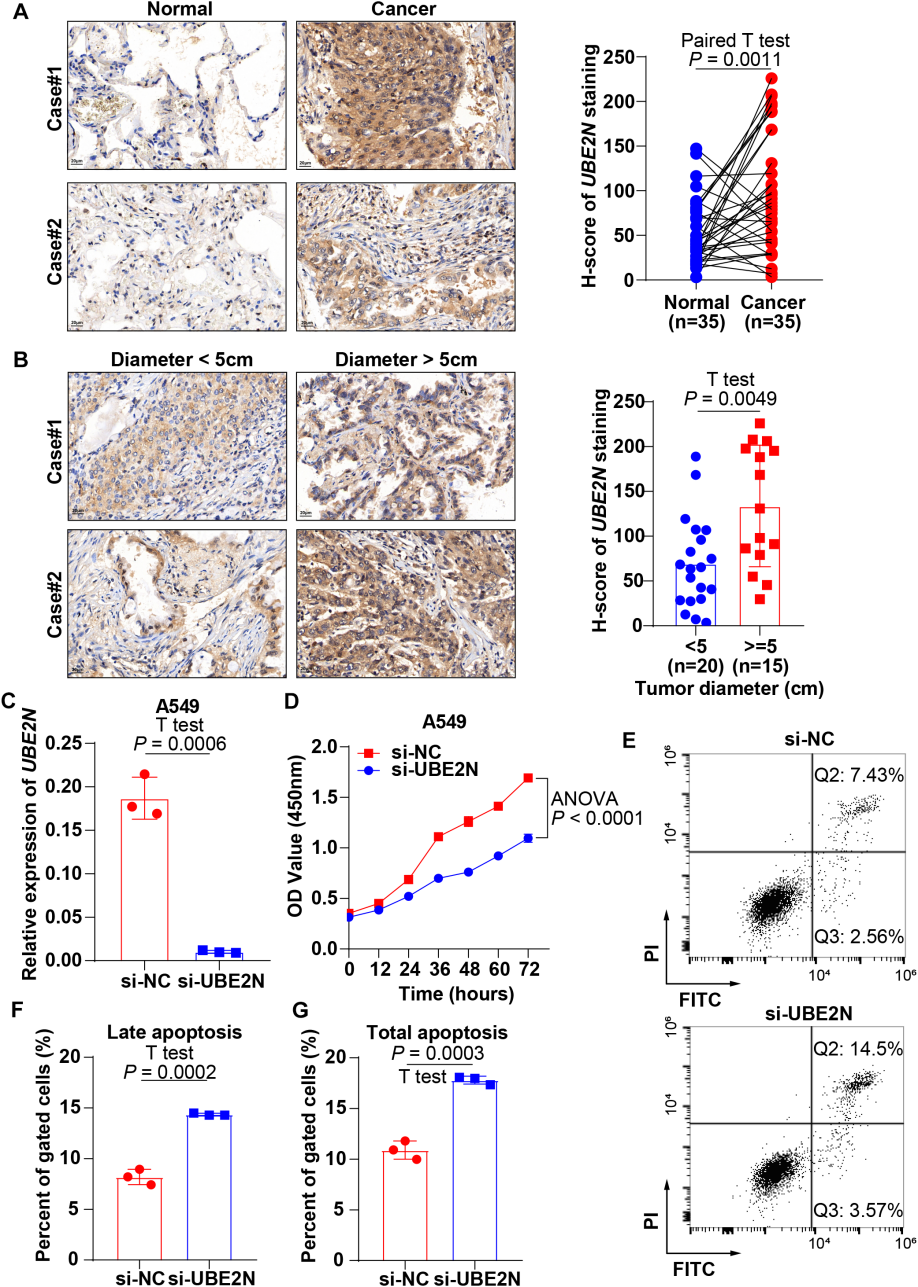
Experimental validation of the expression profile and tumor biology of UBE2N. **(A)** IHC sections reveal high expression of UBE2N in LUAD tissues. **(B)** IHC sections reveal a correlation between UBE2N expression and tumor diameter. **(C)** Knockdown of UBE2N expression with siRNA. **(D)** CCK-8 assay to detect the effect of UBE2N on the proliferative viability of A549 cells. **(E–G)** Experimental detection of the impact of UBE2N on apoptosis in A549 cells.

We validated the expression and biological function of UBE2N in LUAD by pathological assays based on tissue microarrays and cellular experiments. IHC analysis revealed significantly elevated UBE2N expression in LUAD tissues versus paired normal tissues (**Figure 9A**). IHC slices from public databases also demonstrate this pattern (**Supplementary Figure S5**). Furthermore, tumors exceeding 5 cm in diameter also demonstrated significantly elevated UBE2N expression compared to smaller tumors, indicating the oncogenic role of UBE2N (**Figure 9B**). UBE2N knockdown efficiency was validated in A549 cells, with siRNA-mediated silencing reducing UBE2N expression by 75% compared to the negative control (**Figure 9C**). Knockdown of UBE2N significantly impaired cell proliferation, with the proliferative capacity of UBE2N-depleted cells being progressively suppressed over time (**Figure 9D**). Knockdown of UBE2N significantly enhanced apoptosis in A549 cells. Compared to the negative control, UBE2N-knockdown cells exhibited a marked increase in late apoptosis and a modest rise in early apoptosis (**Figures 9E–G**). Consequently, the total apoptotic cell population was significantly increased after knockdown of UBE2N (**Figures 9E–G**). These data nominate UBE2N as both a prognostic biomarker for LUAD aggressiveness and a therapeutic target to disrupt oncogenic resilience.

## 4 Discussion

LUAD represents a significant global health burden with substantial impacts on patient outcomes. Persistent challenges in early-stage LUAD diagnosis stem largely from nonspecific clinical presentation and insufficiently sensitive biomarkers (54). Developing personalized, risk-stratified screening strategies is essential for the early identification of LUAD. In addition, ICB therapy has revolutionized the oncology treatment paradigm, offering novel therapeutic options for patients with refractory, metastatic, or advanced-stage malignancies while demonstrating synergistic effects when combined with chemotherapy or radiotherapy, leading to significantly improved clinical outcomes (55). However, current ICB approaches face substantial challenges, including limited response rates and imperfect predictive biomarkers (9, 55, 56). Systematic multi-omics data mining is critical for the discovery of novel biomarkers with enhanced predictive utility for patient prognosis and therapeutic responsiveness, ultimately enabling precise patient stratification and personalized therapeutic strategies.

In this study, we screened key prognostic genes from multiple patient cohorts and anti-viral gene sets. The interaction between the antiviral immune response and cancer immunity has emerged as an important research area (57, 58). Antiviral signaling pathways, especially those mediated by pattern recognition receptors (PRRs) and interferons (IFNs), not only serve as important intrinsic immune barriers but also profoundly regulate antitumor immunity through mechanisms such as viral mimicry and modulation of immune cell function (59–62). Through multi-cohort prognostic screening and differential analysis, we identified the anti-viral related gene UBE2N as a robust prognostic predictor of LUAD, and high expression of UBE2N could suggest pathological progression and poor prognosis in LUAD patients.

Reportedly, elevated expression of UBE2N was observed in several cancer types, such as prostate cancer, liver cancer, ovarian cancer, and colorectal cancer (63–66). In prostate cancer, the high expression of UBE2N predicted a poor prognosis for patients (63). UBE2N could promote cell survival and glycolysis by activating the Wnt/β-catenin signal in prostate carcinoma. UBE2N could also enhance tumor cell survival and promote ovarian cancer progression and paclitaxel resistance by modulating the Fos/p53 pathway (67). Furthermore, in acute lymphoblastic leukemia models, UBE2N may be a key node of oncogenic immune signaling, as blocking its mediated ubiquitination of innate immune molecules can inhibit its oncogenic function (68). Collectively, these cross-cancer comparisons reveal that role of UBE2N in promoting malignancy might be conserved, but the signaling pathways, cancer hallmarks, and TME interactions it modulates are microenvironmentally and biologically adapted. In the present study, we confirmed the high expression of UBE2N in LUAD and its ability to suggest a poor prognosis for patients in multiple datasets and dimensions, which is in line with a recent study observing the high UBE2N expression suggesting a poor prognosis for LUAD patients (69). However, the links between tumor microenvironment, immune evasion and UBE2N-mediated tumor progression have not yet been adequately revealed. This study not only revealed the association of UBE2N expression with tumor clinicopathological progression but also shed further light on the role and potential of UBE2N from the perspective of tumor immunity and therapeutics, and suggested that UBE2N could serve as a robust biomarker for tumor progression and therapeutic susceptibility.

The enrichment of genes associated with UBE2N expression in pathways related to metabolism and DNA repair provides a plausible mechanism for its role in promoting tumor proliferation and progression (63, 70). High UBE2N expression may lead to increased metabolic activity and enhanced DNA repair capacity, enabling tumors to survive and thrive under conditions of stress and damage (71). The contrasting enrichment patterns of pathways between high and low UBE2N expression tumors offer intriguing insights into the possible mechanisms by which UBE2N regulates tumor progression and immune response (72). The association of high UBE2N expression with pathways related to metabolism and DNA repair suggests a pro-tumorigenic role, while the enrichment of immune-related pathways in low UBE2N expression tumors suggests a potential link between UBE2N expression and immune evasion.

TME is a complex environment composed of tumor cells, immune cells, blood vessels, fibroblasts, extracellular matrix, and a variety of soluble factors (73, 74). These factors interact with each other to regulate cancer growth, invasion, metastasis, and response to therapy. UBE2N may play a crucial role in regulating the TME reshaping. The increased infiltration of neutrophils, γδT cells, and Th2 cells in high-UBE2N tumors may promote the formation of a pro-inflammatory and immunosuppressive microenvironment, facilitating cancer growth and progression. Conversely, in tumors with low UBE2N expression, increased infiltration of B cells, CD8^+^ T cells, and dendritic cells was observed. These cells are capable of directly or indirectly eliminating tumor cells across numerous cancer types (75–78). In addition, we observed that UBE2N might regulate the chemokine-MDSC recruitment, which in turn affects effector T cell infiltration and function. For example, CXCL8-mediated recruitment of myeloid cells limits tumor infiltration of effector T cells and shapes the immunosuppressive TME, thereby hampering cancer immunotherapy (79, 80). The lower levels of stromal components, immune cell infiltration, and comprehensive stromal-immune score in high UBE2N expression tumors further support the hypothesis that UBE2N suppresses the infiltration of effector immune cells into the TME. The association of high UBE2N expression with poor response to immunotherapy suggests that UBE2N may contribute to immune evasion via mechanisms such as shaping an immunosuppressive TME and inhibiting antigen presentation. Previous studies have reported that E2 family member UBE2J1 mediated the ubiquitination and degradation of misfolded MHC-1 heavy chains (81). Ubc9 regulated MHC-II expression in a SUMOylation-dependent manner (82). So, we hypothesize that UBE2N may suppress antigen presentation through ubiquitination-mediated regulation of key regulators in antigen processing pathways. Targeting UBE2N could potentially overcome these barriers and improve the immunotherapy efficacy in LUAD patients.

We identified three potential therapeutic agents targeting UBE2N: genistein, GSK-1059615, and 3-deazaadenosine. Existing studies report that genistein suppresses lung cancer by downregulating the anti-apoptotic factor Bcl-2, upregulating the pro-apoptotic factor Bax, inhibiting proliferation, and inducing apoptosis in lung adenocarcinoma A549 cells, consistent with phenotypes generated by UBE2N inhibition in this study (83, 84); GSK-1059615, as a PI3K and mTOR inhibitor, inhibits tumor cell growth in feline injection-site sarcoma (FISS), though its role in lung cancer remains unelucidated (85, 86); 3-deazaadenosine, functioning as a methylation inhibitor, modulates gene expression through RNA methylation regulation—while its roles in tumorigenesis have been partially explored, its specific mechanisms and effects in lung cancer require further clarification (53).

Some limitations also exist in this study. First, the present study merely focuses on the role of UBE2N in LUAD, while its role in LUSC and other cancer types remains unaddressed. Additionally, the molecular mechanisms underlying how UBE2N affects cancer hallmarks and TME remodeling require further exploration in future studies, and advanced techniques such as proteomics and functional genomics could be employed better to understand the specific functions of UBE2N in cancer. Moreover, the study has inherent limitations including cohort biases from non-random sampling and restricted causal inference due to its retrospective design, which necessitate prospective validation through multi-center cohort studies, longitudinal sampling, and interventional trials; otherwise, the clinical applicability of its findings remains theoretical.

In summary, this work presented UBE2N as a novel prognostic biomarker of LUAD, with its expression levels significantly associated with patient survival, disease progression, hallmark cancer pathways, TME characteristics, and therapeutic response.

## Data Availability

The original contributions presented in the study are included in the article/**Supplementary Material**. Further inquiries can be directed to the corresponding author/s.

## Supplementary material

Supplementary Figure 1 Pan-cancer expression analysis of UBE2N. **(A)** Differential expression of UBE2N in pan-cancer and paracancerous tissues. **(B)** Differential expression of UBE2N in pan-cancer and paired paracancerous tissues.

Supplementary Figure 2 Clinicopathologic correlation and prognostic nomogram of UBE2N. **(A, B)** Association between UBE2N expression and tumor T-stage in GSE48465 **(A)** and GSE50081 cohorts **(B)**. **(C)** Comprehensive prognostic nomogram based on UBE2N expression and clinicopathologic indicators.

Supplementary Figure 3 Differences in GSVA scores across UBE2N expression groups. **(A)** GSVA scores of cell cycle and DNA damage repair-related gene sets. **(B)** GSVA scores of immune function gene sets. **(C)** Correlation of UBE2N expression with common chemokines and cytokines. **(D, E)** Correlation of UBE2N expression with CXCL8 expression **(D)** and MDSC signature scores **(E)**.

Supplementary Figure 4 ROC curves for assessing the performance of UBE2N in predicting immunotherapy response. The results of **(A–C)** were based on GSE126044, BMS038, and Gide_2019 datasets, respectively.

Supplementary Figure 5 Validation of UBE2N expression profile in LUAD based on IHC data from the HPA database. **(A)** IHC images of UBE2N expression in LUAD and normal lung tissues.

Supplementary Table 1 Information on public datasets used in this study.

Supplementary Table 2 Information on public gene sets used in this study.

Supplementary Table 3 Primers and siRNA sequences.

Supplementary Table 4 Information on candidate compounds.
