# Supplementary figures and images for: UBE2N as a novel prognostic and therapeutic biomarker of lung adenocarcinoma
Source: Front Immunol. 2025 Aug 11;16:1636503. doi: 10.3389/fimmu.2025.1636503 (PMC12375651; doi:10.3389/fimmu.2025.1636503)

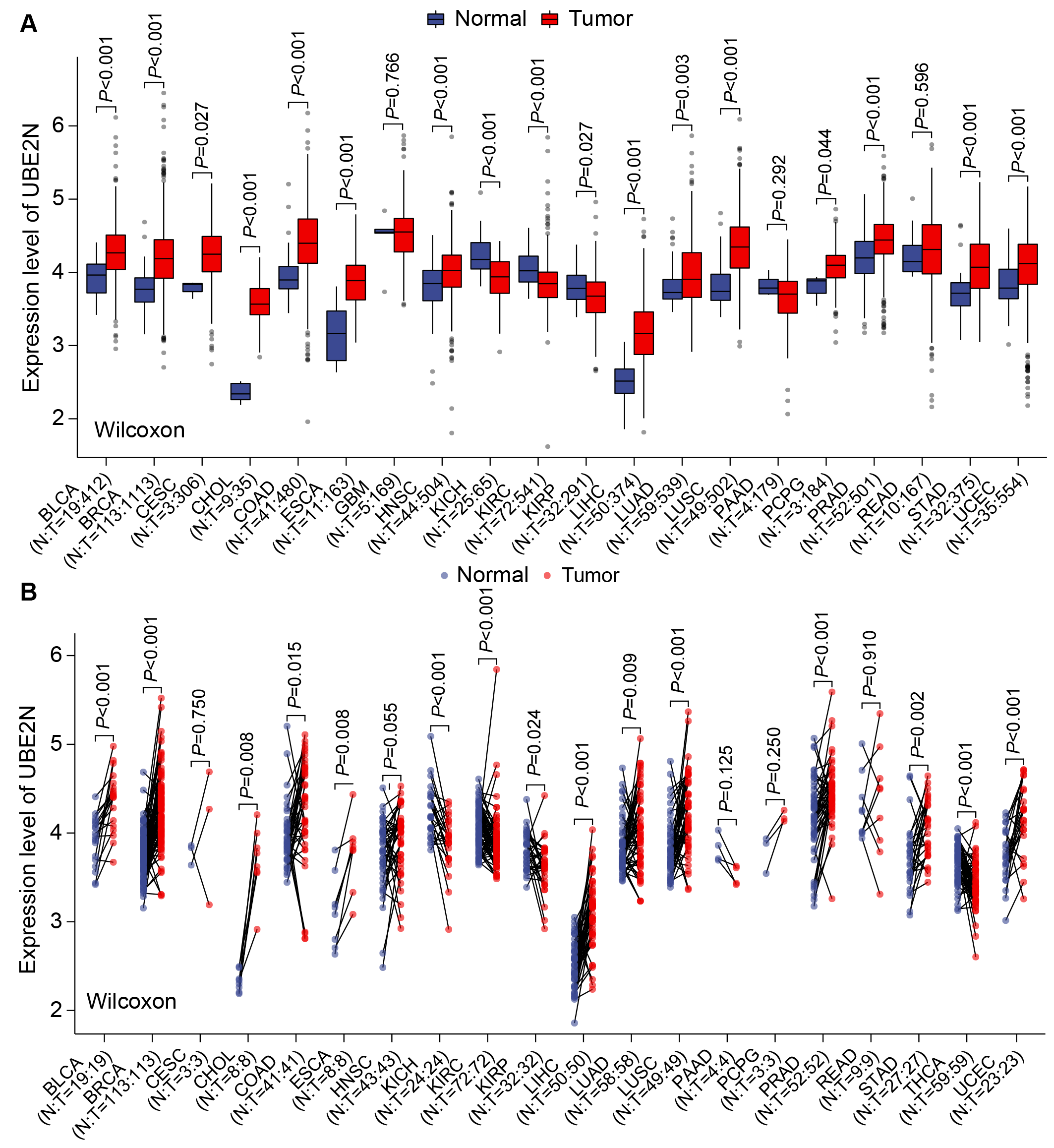

Supplement: Supplementary Figure 1 — Pan-cancer expression analysis of UBE2N. (A) Differential expression of UBE2N in pan-cancer and paracancerous tissues. (B) Differential expression of UBE2N in pan-cancer and paired paracancerous tissues. [file Image1.tif]

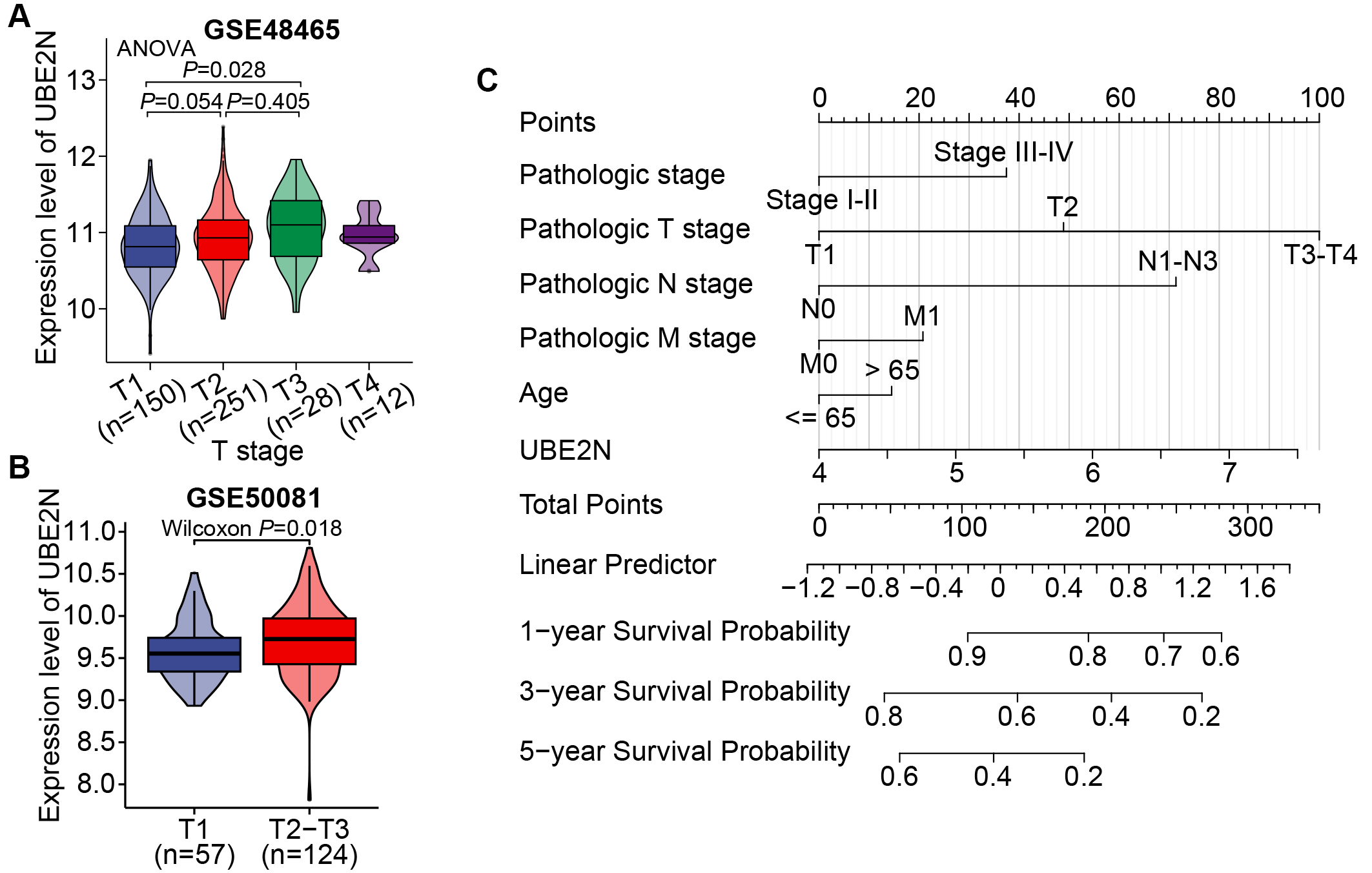

Supplement: Supplementary Figure 2 — Clinicopathologic correlation and prognostic nomogram of UBE2N. (A, B) Association between UBE2N expression and tumor T-stage in GSE48465 (A) and GSE50081 cohorts (B). (C) Comprehensive prognostic nomogram based on UBE2N expression and clinicopathologic indicators. [file Image2.tif]

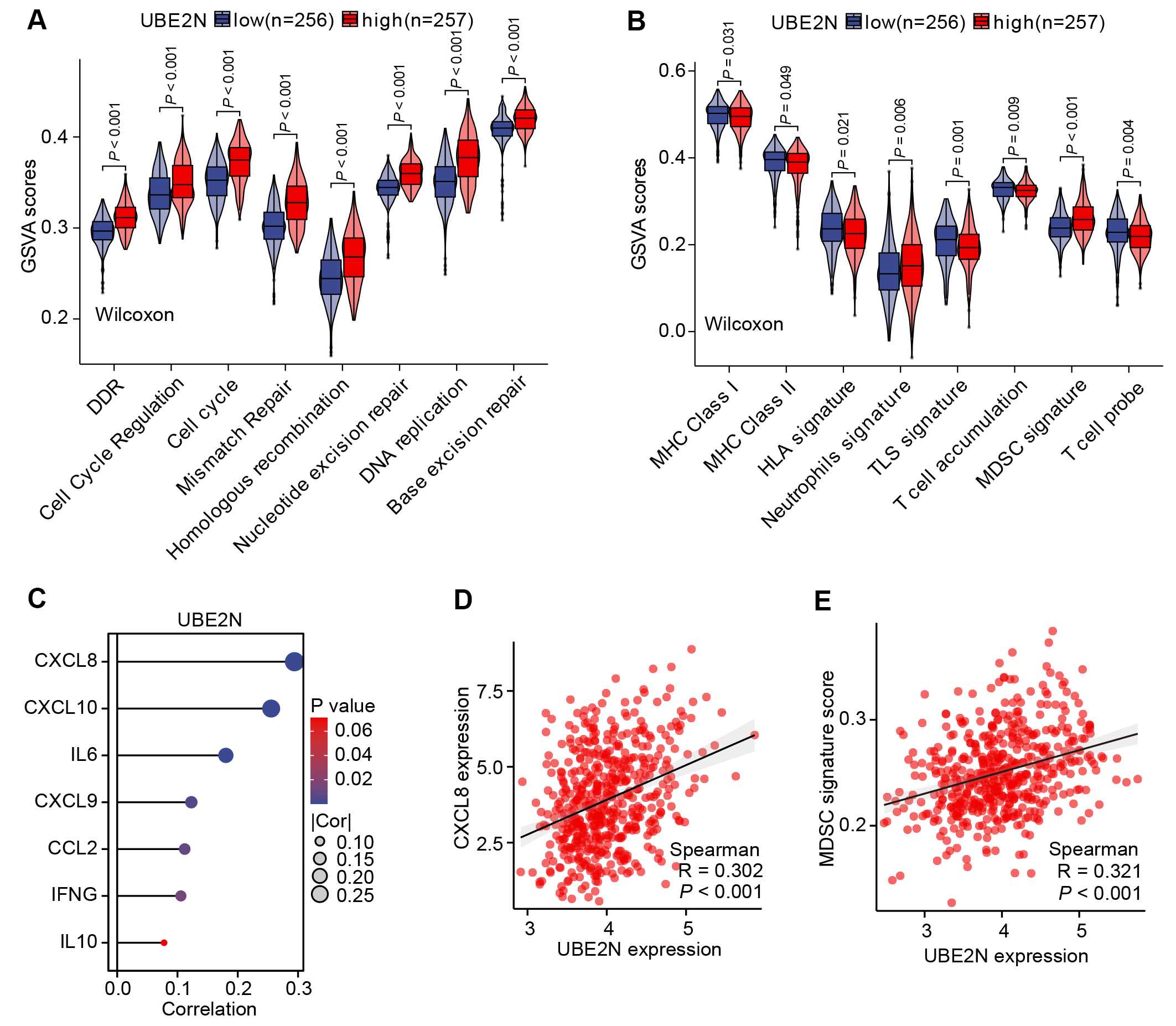

Supplement: Supplementary Figure 3 — Differences in GSVA scores across UBE2N expression groups. (A) GSVA scores of cell cycle and DNA damage repair-related gene sets. (B) GSVA scores of immune function gene sets. (C) Correlation of UBE2N expression with common chemokines and cytokines. (D, E) Correlation of UBE2N expression with CXCL8 expression (D) and MDSC signature scores (E). [file Image3.tif]

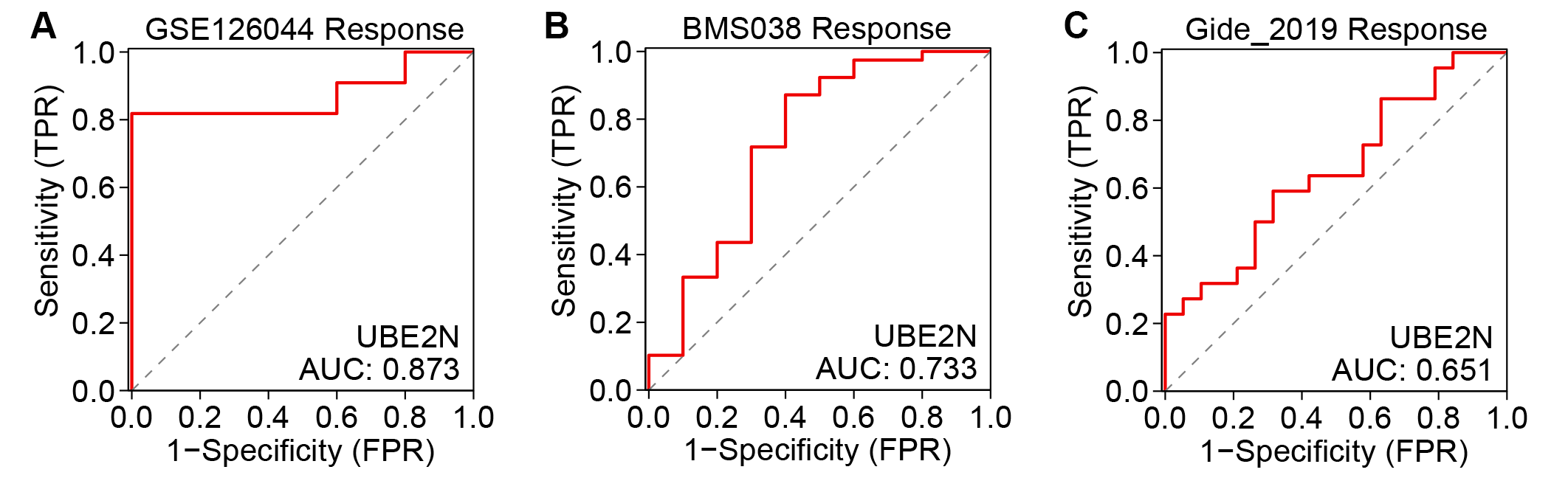

Supplement: Supplementary Figure 4 — ROC curves for assessing the performance of UBE2N in predicting immunotherapy response. The results of (A–C) were based on GSE126044, BMS038, and Gide_2019 datasets, respectively. [file Image4.tif]

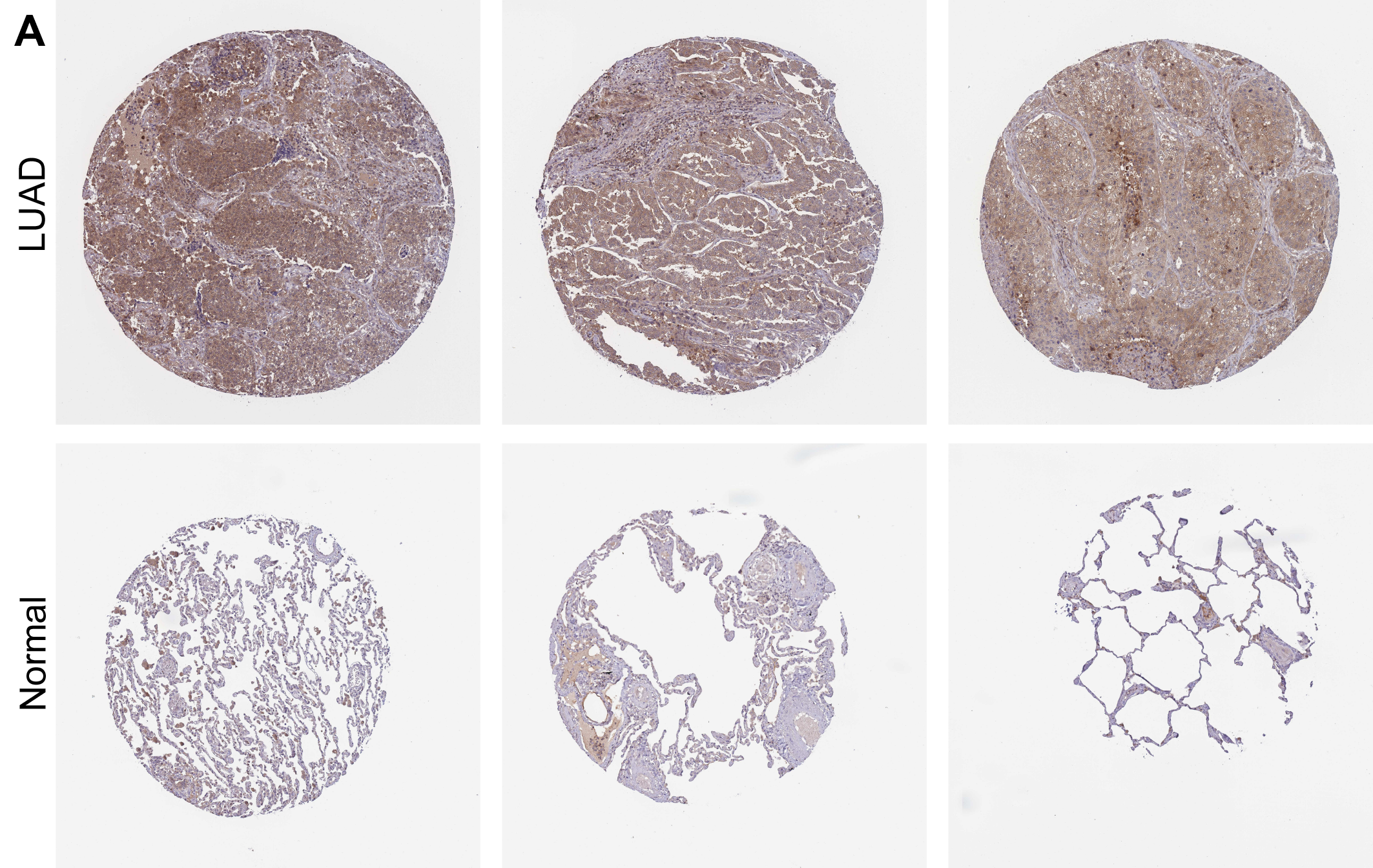

Supplement: Supplementary Figure 5 — Validation of UBE2N expression profile in LUAD based on IHC data from the HPA database. (A) IHC images of UBE2N expression in LUAD and normal lung tissues. [file Image5.tif]
