# Supplementary material for: UBE2N as a novel prognostic and therapeutic biomarker of lung adenocarcinoma
Source: Front Immunol. 2025 Aug 11;16:1636503. doi: 10.3389/fimmu.2025.1636503 (PMC12375651; doi:10.3389/fimmu.2025.1636503)
Supplement: Supplementary Table 1 — Information on public datasets used in this study. [file Table1.docx]

**Supplementary materials**

**Supplementary Table S1.** Information on public datasets used in this study

| ID | Application |
| --- | --- |
| TCGA-LUAD | Survival analysis; Clinicopathological relevance |
| GSE31210 | Survival analysis; Clinicopathological relevance |
| GSE30219 | Survival analysis; Clinicopathological relevance |
| GSE3141 | Survival analysis |
| GSE50081 | Survival analysis |
| GSE19188 | Survival analysis |
| GSE48465 | Clinicopathological relevance |
| GSE78220 | Immunotherapy response |
| GSE91061 | Immunotherapy response |
| Phs000452 | Immunotherapy response |
| GSE126044 | Immunotherapy response |
| Gide_2019 | Immunotherapy response |
| PMID29301958 | In vitro CRISPR screen |
| PMID33589424 | In vitro CRISPR screen |
| PMID36151395 | In vivo CRISPR screen |

**Supplementary Table S2.** Information on public gene sets used in this study

| ID | Application | Source |
| --- | --- | --- |
| KEGG pathway gene sets | Functional enrichment analysis | [www.gsea-msigdb.org](http://www.gsea-msigdb.org/) |
| Gene Ontology gene sets | Functional enrichment analysis | www.gsea-msigdb.org |
| Immune cell markers | Immune cell infiltration analysis | 10.1016/j.immuni.2013.10.003 |
| DNA damage repair-related gene sets | GSVA | IOBR package: 10.1016/j.crmeth.2024.100910 |
| Cell cycle-related gene sets | GSVA | IOBR package: 10.1016/j.crmeth.2024.100910 |
| Immune function gene sets | GSVA | IOBR package: 10.1016/j.crmeth.2024.100910 |

**Supplementary Table S3.** Primers and siRNA sequences

| Name | Target | Direction | Sequences |
| --- | --- | --- | --- |
| qPCR primers | UBE2N | Forward | CCCCGCAGGATCATCAAGG |
| qPCR primers | UBE2N | Reverse | CCCTCCCTCAAAGGGGGAA |
| qPCR primers | GAPDH | Forward | GGAGCGAGATCCCTCCAAAAT |
| qPCR primers | GAPDH | Reverse | GGCTGTTGTCATACTTCTCATGG |
| siRNA | UBE2N | Sense | GGAAGAAUAUGUUUAGAUAUU |
| siRNA | UBE2N | Anti-sense | UAUCUAAACAUAUUCUUCCCA |

**Supplementary Table S4.** Information on candidate compounds

| PubChem ID | Compound | Molecular Formula |  | Link |
| --- | --- | --- | --- | --- |
| 5280961 | Genistein | C_15_H_10_O_5_ |  | <https://pubchem.ncbi.nlm.nih.gov/compound/5280961> |
| 23582824 | GSK-1059615 | C_18_H_11_N_3_O_2_S |  | <https://pubchem.ncbi.nlm.nih.gov/compound/23582824> |
| 23190 | 3-Deazaadenosine | C_11_H_14_N_4_O_4_ |  | <https://pubchem.ncbi.nlm.nih.gov/compound/23190> |
